# Supplementary material for: Modeling glioblastoma heterogeneity as a dynamic network of cell states
Source: Mol Syst Biol. 2021 Sep 16;17(9):e10105. doi: 10.15252/msb.202010105 (PMC8444284; doi:10.15252/msb.202010105)
Supplement: Supplementary file 6 — Source Data for Figure 5 [file MSB-17-e10105-s004.zip › Figure5A_sourcedata/GSEA_3017/nosigdb_stateD.GseaPreranked.1622795010397/RICHARDS_ZHONG_NATURE_2018_NPCS_UPREG.html]

Details for gene set RICHARDS\_ZHONG\_NATURE\_2018\_NPCS\_UPREG[GSEA]

|  || Dataset | state23017 |
| Phenotype | NoPhenotypeAvailable |
| Upregulated in class | na\_neg |
| GeneSet | RICHARDS\_ZHONG\_NATURE\_2018\_NPCS\_UPREG |
| Enrichment Score (ES) | -0.5851479 |
| Normalized Enrichment Score (NES) | -3.0708873 |
| Nominal p-value | 0.0 |
| FDR q-value | 0.0 |
| FWER p-Value | 0.0 |
Table: GSEA Results Summary

  

Fig 1: Enrichment plot: RICHARDS\_ZHONG\_NATURE\_2018\_NPCS\_UPREG      
 Profile of the Running ES Score & Positions of GeneSet Members on the Rank Ordered List

  

| PROBE | GENE SYMBOL | GENE\_TITLE | RANK IN GENE LIST | RANK METRIC SCORE | RUNNING ES | CORE ENRICHMENT || 1 | PTTG1 |  |  | 14 | 0.490 | -0.0010 | No |
| 2 | ID4 |  |  | 38 | 0.374 | -0.0118 | No |
| 3 | HSPB1 |  |  | 52 | 0.347 | -0.0150 | No |
| 4 | TGIF1 |  |  | 55 | 0.343 | -0.0095 | No |
| 5 | VIM |  |  | 75 | 0.314 | -0.0183 | No |
| 6 | HMGB3 |  |  | 80 | 0.311 | -0.0150 | No |
| 7 | LDHA |  |  | 92 | 0.297 | -0.0176 | No |
| 8 | BIRC5 |  |  | 130 | 0.271 | -0.0419 | No |
| 9 | MKI67 |  |  | 206 | -0.254 | -0.0972 | No |
| 10 | CNTLN |  |  | 225 | -0.256 | -0.1064 | No |
| 11 | MCM2 |  |  | 226 | -0.256 | -0.1011 | No |
| 12 | PRR11 |  |  | 361 | -0.269 | -0.2038 | No |
| 13 | UHRF1 |  |  | 438 | -0.278 | -0.2595 | No |
| 14 | APOLD1 |  |  | 476 | -0.282 | -0.2835 | No |
| 15 | BTG3 |  |  | 581 | -0.296 | -0.3614 | No |
| 16 | SPAG5 |  |  | 605 | -0.301 | -0.3737 | No |
| 17 | MCM3 |  |  | 625 | -0.305 | -0.3827 | No |
| 18 | PAX6 |  |  | 629 | -0.307 | -0.3787 | No |
| 19 | PRC1 |  |  | 645 | -0.310 | -0.3843 | No |
| 20 | ZFHX4 |  |  | 656 | -0.311 | -0.3859 | No |
| 21 | PHGDH |  |  | 662 | -0.312 | -0.3833 | No |
| 22 | TMPO |  |  | 688 | -0.317 | -0.3969 | No |
| 23 | KNSTRN |  |  | 690 | -0.317 | -0.3911 | No |
| 24 | WDR34 |  |  | 752 | -0.331 | -0.4335 | No |
| 25 | MCM4 |  |  | 783 | -0.339 | -0.4506 | No |
| 26 | CDC20 |  |  | 793 | -0.341 | -0.4507 | No |
| 27 | H2AFX |  |  | 810 | -0.347 | -0.4564 | No |
| 28 | CENPM |  |  | 818 | -0.349 | -0.4547 | No |
| 29 | MAD2L1 |  |  | 819 | -0.350 | -0.4474 | No |
| 30 | BUB3 |  |  | 826 | -0.351 | -0.4449 | No |
| 31 | CDCA4 |  |  | 870 | -0.363 | -0.4720 | No |
| 32 | CKAP2 |  |  | 900 | -0.375 | -0.4876 | No |
| 33 | TUBB4B |  |  | 916 | -0.380 | -0.4918 | No |
| 34 | DEPDC1B |  |  | 923 | -0.383 | -0.4886 | No |
| 35 | HAT1 |  |  | 933 | -0.387 | -0.4878 | No |
| 36 | KIF11 |  |  | 1003 | -0.416 | -0.5349 | No |
| 37 | GLI3 |  |  | 1016 | -0.424 | -0.5357 | No |
| 38 | TPX2 |  |  | 1034 | -0.437 | -0.5403 | No |
| 39 | KIF22 |  |  | 1051 | -0.446 | -0.5438 | No |
| 40 | SMC2 |  |  | 1062 | -0.452 | -0.5425 | No |
| 41 | ITGB3BP |  |  | 1096 | -0.467 | -0.5594 | No |
| 42 | ZWINT |  |  | 1099 | -0.467 | -0.5512 | No |
| 43 | PCNA |  |  | 1142 | -0.504 | -0.5746 | Yes |
| 44 | CKAP2L |  |  | 1147 | -0.508 | -0.5671 | Yes |
| 45 | NUSAP1 |  |  | 1154 | -0.515 | -0.5612 | Yes |
| 46 | GMNN |  |  | 1155 | -0.516 | -0.5504 | Yes |
| 47 | MIS18BP1 |  |  | 1162 | -0.524 | -0.5442 | Yes |
| 48 | RRM1 |  |  | 1166 | -0.534 | -0.5354 | Yes |
| 49 | ORC6 |  |  | 1179 | -0.551 | -0.5336 | Yes |
| 50 | CENPU |  |  | 1182 | -0.555 | -0.5235 | Yes |
| 51 | KIF2C |  |  | 1183 | -0.558 | -0.5118 | Yes |
| 52 | CDCA3 |  |  | 1187 | -0.563 | -0.5024 | Yes |
| 53 | ASPM |  |  | 1189 | -0.567 | -0.4913 | Yes |
| 54 | FEN1 |  |  | 1191 | -0.569 | -0.4802 | Yes |
| 55 | NUF2 |  |  | 1194 | -0.574 | -0.4698 | Yes |
| 56 | DHFR |  |  | 1197 | -0.576 | -0.4593 | Yes |
| 57 | CKS1B |  |  | 1198 | -0.577 | -0.4472 | Yes |
| 58 | GTSE1 |  |  | 1210 | -0.602 | -0.4435 | Yes |
| 59 | CLSPN |  |  | 1212 | -0.605 | -0.4316 | Yes |
| 60 | KIF15 |  |  | 1213 | -0.610 | -0.4188 | Yes |
| 61 | CENPK |  |  | 1220 | -0.619 | -0.4106 | Yes |
| 62 | C21orf58 |  |  | 1222 | -0.626 | -0.3983 | Yes |
| 63 | DUT |  |  | 1223 | -0.633 | -0.3850 | Yes |
| 64 | SMC4 |  |  | 1224 | -0.638 | -0.3716 | Yes |
| 65 | CKS2 |  |  | 1226 | -0.641 | -0.3590 | Yes |
| 66 | CDCA5 |  |  | 1244 | -0.685 | -0.3583 | Yes |
| 67 | TAGLN2 |  |  | 1247 | -0.698 | -0.3453 | Yes |
| 68 | AURKB |  |  | 1250 | -0.700 | -0.3322 | Yes |
| 69 | MELK |  |  | 1251 | -0.701 | -0.3175 | Yes |
| 70 | CCNA2 |  |  | 1255 | -0.716 | -0.3049 | Yes |
| 71 | UBE2T |  |  | 1256 | -0.719 | -0.2898 | Yes |
| 72 | HMGB2 |  |  | 1263 | -0.736 | -0.2792 | Yes |
| 73 | KIF23 |  |  | 1265 | -0.741 | -0.2645 | Yes |
| 74 | TACC3 |  |  | 1268 | -0.751 | -0.2504 | Yes |
| 75 | ATAD2 |  |  | 1275 | -0.775 | -0.2389 | Yes |
| 76 | NDC80 |  |  | 1278 | -0.810 | -0.2236 | Yes |
| 77 | HJURP |  |  | 1290 | -0.897 | -0.2136 | Yes |
| 78 | SPC25 |  |  | 1294 | -0.932 | -0.1965 | Yes |
| 79 | RRM2 |  |  | 1295 | -0.933 | -0.1769 | Yes |
| 80 | KPNA2 |  |  | 1297 | -0.972 | -0.1573 | Yes |
| 81 | FBXO5 |  |  | 1301 | -0.992 | -0.1389 | Yes |
| 82 | CDK1 |  |  | 1311 | -1.433 | -0.1161 | Yes |
| 83 | UBE2C |  |  | 1312 | -1.466 | -0.0853 | Yes |
| 84 | TOP2A |  |  | 1313 | -1.528 | -0.0533 | Yes |
| 85 | HIST1H4C |  |  | 1320 | -2.769 | 0.0000 | Yes |
Table: GSEA details [plain text format]

  

Fig 2: RICHARDS\_ZHONG\_NATURE\_2018\_NPCS\_UPREG: Random ES distribution      
 Gene set null distribution of ES for **RICHARDS\_ZHONG\_NATURE\_2018\_NPCS\_UPREG**
